# Psychological barriers in children: an exploratory study on Dengue transmission using an adapted DIPB scale

**DOI:** 10.3389/fpsyg.2024.1412856

**Published:** 2024-10-30

**Authors:** Pedro Schimmelpfeng, Luiz Gonzaga Lapa, Claudia Marcia Lyra Pato

**Affiliations:** School of Education, University of Brasília, Brasília, Brazil

**Keywords:** pro-environmental behavior, environmental education, dragons of inaction, Dengue fever, *Aedes aegypti*, psychological barriers

## Abstract

Dengue is an arboviral infection found in tropical and subtropical regions transmitted by hematophagous mosquitoes from the genus *Aedes* spp. and responsible for millions of cases every year. Public campaigns and educational curriculum are designed to educate people, including children. However, what has been reported is that many decide not to follow these guidelines, even though they allegedly know what has to be done. To understand this phenomenon, this study aims to identify psychological barriers behind the adoption of pro-environmental behaviors that seek to reduce *Aedes aegypti*’s population. For that, middle school students participated on two studies responsible for (1) adapting the Dragons of Inaction Psychological Barrier (DIPB) scale to the target group (*n* = 150) and then (2) testing it on a larger group (*n* = 449). In the exploratory factor analysis, Bartlett correlation (*p* < 0.001), Cronbach’s alpha (0.83), and KMO analysis (overall MSA = 0.84) showed that data was suited for factor analysis. Five factors were retained by Kaiser Criterion and scree test (i.e., Conflicting goals and unnecessary changes—*α* 0.76, Interpersonal relations—*α* 0.72, Conflicting goals and lacking knowledge—*α* 0.58, Tokenism—α 0.73, and Tokenism toward the government—*α* 0.66). After that, the scale was tested across 11 different schools, where students also answered a questionnaire about the mosquito. Results suggested that the factors Conflicting goals and lacking knowledge and Tokenism toward the government presented a higher level of agreement for all students (means: 2.6 and 2.12 out of five, respectively). Those who scored higher in the mosquito’s questionnaire had factors Conflicting goals and unnecessary change and Interpersonal relations inhibited when compared to others (*p* < 0.05). These results suggests that future educational campaigns should build different actions that focuses on addressing both internal and external factors, creating a mosaic of projects, with different goals, each aiming different environmental challenges.

## Introduction

Dengue is an arboviral infection found in tropical and subtropical regions, causing 400 million cases and 22,000 deaths worldwide every year (Hosseini et al., 2018; Roy and Bhattacharjee, 2021). In Brazil, *A. aegypti* and Dengue reemerged over 40 years ago (Teixeira et al., 2009), and while in 2022 1.393.684 probable cases were identified, more than 5 million cases were reported for the first semester of 2024 already (Brazil, 2023, 2024).

Arboviruses are those transmitted by hematophagous arthropods and, in this case, by mosquitoes from the genus *Aedes* spp., where *Aedes aegypti* and *Aedes albopictus* are the most common ones (Kraemer et al., 2015). The mosquito’s female feeds on the blood from its host to mature its eggs, which are later going to be deposited on stagnant water. It is during the feeding moment that the insect injects its saliva, which may contain the Dengue virus and others (i.e., Chikungunya, Zika and Yellow fever viruses) (Junior et al., 2015; Vu et al., 2017).

In nature, these insects usually use water that accumulates in some plant parts, like in bromeliads, for example. However, in urban environments, they find a larger number of options, like plates of plants, empty bottles, open water tanks, poorly discarded tires and others. These objects can easily accumulate water from the rain and become adequate spots for oviposition.

To reduce the mosquitos’ population, a combination of actions from the government and general public are necessary (i.e., environmental interventions) (Zara et al., 2016). From the population’s perspective, to avoid any stagnant water in their houses and appropriate trash disposal are the most effective interventions. These reduce entomological indicators, involve few resources, and are easy to implement (Tortosa-La Osa et al., 2022).

The set of behaviors necessary for these interventions are called Pro-Environmental Behaviors (PEB’s) (Iglesias et al., 2014; Karp, 1996; Lange and Dewitte, 2019). To achieve this set, multiple variable are taken into consideration, split into personal and context levels of approach (Corral-Verdugo and Pinheiro, 1999; Schultz and Kaiser, 2012), internal and external factors (Kollmuss and Agyeman, 2010) and cultural aspects of societies (Tam and Chan, 2017).

Nevertheless, when considering that PEB’s cannot be imposed, what can be achieved is the establishment of a positive attitude toward a specific behavior, that is built alongside a strong system of values and beliefs (Best and Mayerl, 2013; Corraliza and Berenguer, 2000; Pato and Delabrida, 2019; Pato and Tamayo, 2006). In order to better understand this phenomenon, many articles have discussed it in recent years using a wide variety of groups and scenarios, like celebrities endorsement on the use of single-use plastics (Ho et al., 2022), how policy makers can encourage PEB amongst their target audiences (Lucas et al., 2008), meat consumption (Graves and Roelich, 2021), and others (Grilli and Curtis, 2021; Yuriev et al., 2018).

However, despite some positive results, what is seen in recent literature is a gap between presenting a positive attitude toward PEBs and actually manifesting them (Gaspar, 2013; Kollmuss and Agyeman, 2010; Quimby and Angelique, 2011). These psychological barriers are explained by a number of factors, which have been named Dragons of Inaction (Gifford, 2011).

Most recently, a study tried to develop a scale to assess factors involved in the lack of action toward various environmental problems (Lacroix et al., 2019) [i.e., (1) Change unnecessary, (2) Conflicting goals and aspirations, (3) Interpersonal relations, (4) Lacking knowledge, and (5) Tokenism]. This scale, called the Dragons of Inaction Psychological Barriers’ scale (DIPB scale) has been validated and tested by undergrad students in Canada (Lacroix et al., 2019), United States (Mouchrek et al., 2022) and Colombia (Gutiérrez et al., 2022), but it has not been validated in Brazilian territory or a younger group of people. Factors within a scale are variables that are being tested for a specific barrier.

Regarding *A. aegypti*, the population is usually instigated by various educational programs accessed over the television and activities in schools, parks, and public spaces. Not only that, but sanitary agents also visit residences to check for possible sites of oviposition and to answer questions by the public. Although educational campaigns have previously shown a positive correlation between knowledge and preventive practices (Brusich et al., 2015; Yasmeen et al., 2015), there is still a group of people who choose not to take part on it (Caregnato et al., 2008; Chiaravalloti Neto, 1997; Chiaravalloti Neto et al., 1998; Chiaravalloti et al., 2002; Claro et al., 2004). At the same time, a more recent review recognizes that educational campaigns are essential to reduce breeding sites and interrupt disease transmission, but questions the quality of evidence, considering a multi modal approach (Bouzid et al., 2016). To propose new insights on how educational campaigns can be more effective to prevent mosquitoes’ proliferation, this study aims to identify possible psychological barriers behind the adoption of pro-environmental behaviors expected to reduce *Aedes aegypti*’s population.

## Materials and methods

This is an exploratory study where two questionnaires were used to create enough data for a quantitative analysis. This article is divided than into two studies, one for validating the Dragons of Inaction Psychological Barriers scale and the second for verifying possible barriers and assimilation of concepts by students from different schools.

### Data collection and participants

For both studies, 11 public schools from the city of Volta Redonda, Brazil, all urban, were visited. Then, seventh grade students (age’s mean 12.52 years old, SD = 0.866) were selected to answer the questionnaires during the year of 2023. The city is located in the inner part of Rio de Janeiro’s state, southeast of the country, and many educational campaigns have been made over the last years to educate its population on the expected PEB’s to control the mosquito. These campaigns are mostly run through the radio and television, with specific educational actions also happening in city events, many targeted to children, and by public environmental agents that visit houses and other facilities to check for contaminated spots.

School’s infrastructure varied depending on the city’s regions, but a correlation between it and dengue’s knowledge was not expected, considering that the majority of educational campaigns on this topic happens outside the school environment. Nevertheless, it is part of the 7th grade Brazilian science curriculum to discuss health indicators in a community, considering access to basic sanitation and common illness, such as Dengue fever. Because of these aspects, it is expected that children around that age and grade already have the knowledge expected to inhibit mosquitoes’ proliferation.

Thus, after the initial contact with each school’s principal, all schools were visited to explain what the research was about and invite students to participate. They were handed a form to be sent to their parents, explaining what the questionnaires were about, research’s relevance, and contact information. Only students that turned in those documents, signed by one of their parents or legal caretakers, and agreed to participate in this research could answer the questionnaires on the following meeting. This study was approved by the human research ethics committee of the University of Brasilia.

On the agreed day, groups of 10 students were asked to come to the school’s library, where the questionnaires were handed, and instructions were given. After finishing answering it, they were sent back to class. The whole process for each group lasted 30 min approximately.

On the first study, 150 students (81 male and 69 female) were selected to answer the 22-items DIPB scale. These results were used for an Exploratory factor analysis (EFA), and, after making the required adjustments, the DIPB scale and Dengue’s questionnaire were applied to a new set of students following the same criteria (*n* = 449, 213 males and 236 females) (Study 2). To present the results, items were later translated back to the English language again.

### Dragons of inaction psychological barriers scale’s adaptation

Initially, the DIPB scale developed by Lacroix et al. (2019) had to be adapted to the Brazilian perspective. For that, first, three different English teachers translated each of the 22 items from the original scale. Secondly, items were adapted to accommodate to the literacy expected from kids at this age group (12–13 years old). To specify the relatedness to each of the items, a Likert scale-using emojis was used, based on a five point system (Bouranta et al., 2009; Lapa, 2019; Preston and Colman, 2000).

### Dengue’s questionnaire

This questionnaire contained four questions, where each covered one of the major skills expected by the population to prevent mosquito’s reproduction and proliferation. They are: (Item 01) to recognize the different stages of the insect’s life cycle (egg, larvae, pupae, and adult), (Item 02) to associate its different stages to environmental factors (i.e., stagnant water), (Item 03) to recognize different diseases transmitted by the mosquito, and (Item 04) to identify the main prophylactic measures to reduce mosquito’s population.

On item 01, students would have to number the correct sequence that represents the order of stages for the insect’s life cycle (i.e., egg, larvae, pupae, and adult). On items 02, 03, and 04, students would be presented nine different options, where some of them were correct while others were wrong, having to identify, by marking an “X,” the correct ones.

For items 02, 03, and 04, in order to identify false positive results (i.e., students randomly guessing multiple items), options within the items that were marked incorrectly nullified a correct marking. The lowest score for each of those items would be zero, and students that scored maximum points did that by selecting only correct options.

In the end, each student would be graded from 0 to a 100 (i.e., 25 points from each question) based on the answers given. The set of results would then be divided into quartiles for data comparison with the DIPB scale.

## Results

### Study 1—Exploratory Factor Analysis

Principal axis factor analysis with oblimin rotation was used to extract factors. Bartlett correlation test (*p* < 0.001) and KMO analysis (overall MSA = 0.84; meritorious) showed that data was suited for factor analysis and *α*-Cronbach was 0.85. Items that showed low communalities (lower than 0.30) and/or were loaded in more than one factor were eliminated. The Kaiser Criterion (i.e., eigenvalue >1) and scree test suggested retaining five factors, explaining 62.66% of the variation. The results for the EFA proposed the removal of five items and reorganized the five factors (Table 1). From the remaining items, two of them (items 1 and 17) had low communalities but were retained due to its loadings within their correspondent factors.

**Table 1:** Exploratory factor analysis—standard matrix.

Item	Factors				
	1	2	3	4	5
7	0.684				
10	0.620				
8	0.581				
2	0.541				
1	0.454				
14		0.778			
11		0.590			
13		0.546			
23			−0.872		
24			−0.561		
18			−0.397		
9				0.646	
17				0.511	
15				0.439	
20					−884
21					−0.650
19					−0.391

Extraction method: principal axis factor analysis. Rotation method: Oblimin and Kaiser normalization. Converged rotation after 11 iterations.

**Figure 1:**
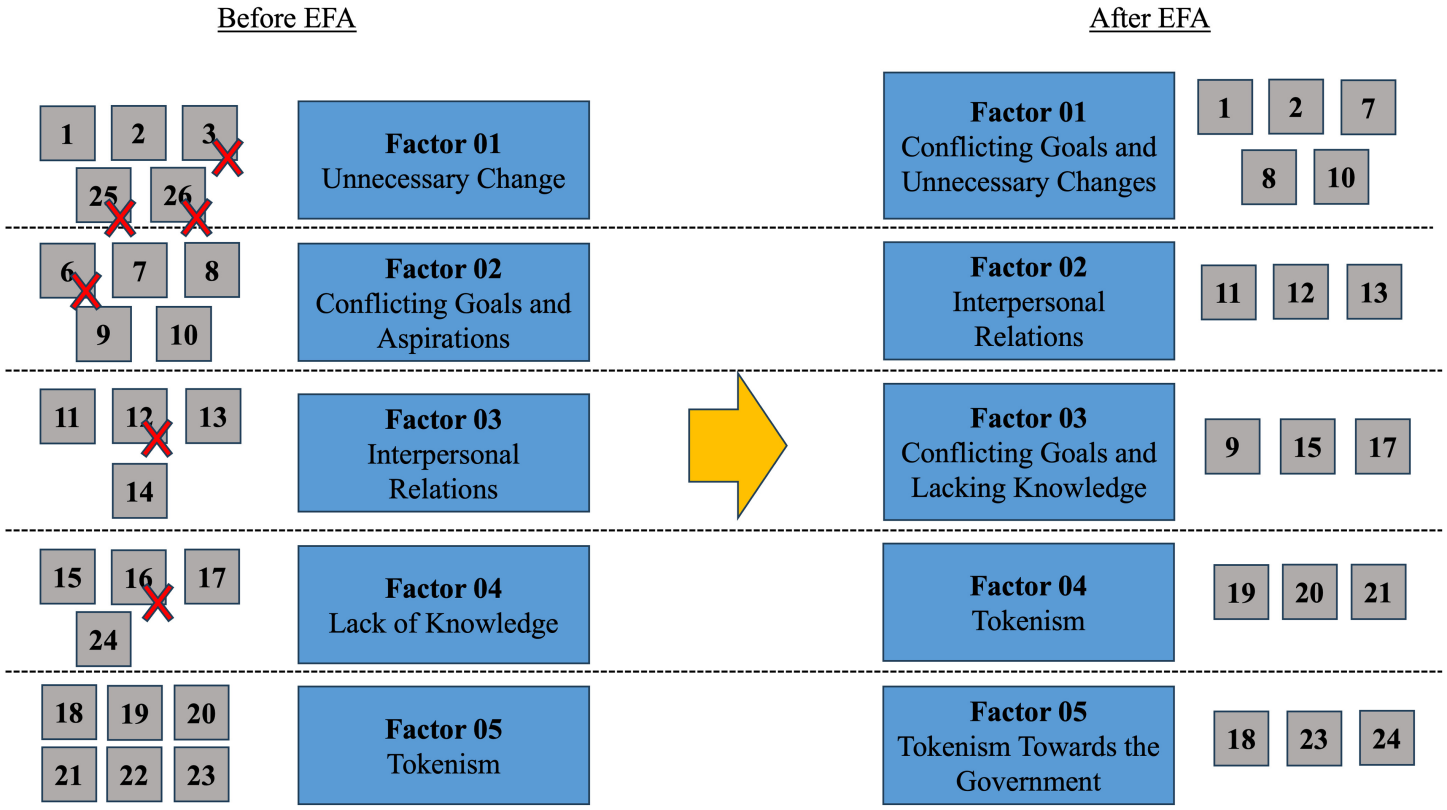
New arrangement for the DIPB scale after exploratory factor analysis (EFA). On the left, grey boxes represent items within each factor in the original DIPB scale, while on the right is shown the new configuration after EFA. Blue boxes represent factor’s previous names (left) and adjusted names (right). The red crosses show items that were eliminated during EFA.

The new factor distribution rearranged the 17 items into five different factors, where factor 01 contained five items and other factors three items (Table 2; Figure 1). Names of each factor were altered to accommodate the proposed changes.

**Table 2:** Original DIPB scale and its adapted version containing Cronbach’s alpha (α) for its factors.

Original—Psychological barrier factors and items		
Factor 1: Change unnecessary		
1	There’s not much point in me making this change because I feel confident that technological innovators will solve environmental problems.	
2	Humans are powerless when it comes to saving the Earth, so there is no need to change.	
3	These problems are so far in the future, so there is no need to act.	
25	There’s a need for change because I believe that a serious environmental problem exists.	
26	What happens at the industrial level makes my changing insignificant.	
Factor 2: Conflicting goals and aspirations		
6	Making this change would interfere too much with my other goals in life.	
7	I’m concerned that this change will take up too much of my time.	
8	I can’t change because I’m invested in my current lifestyle.	
9	These issues are important to me but it’s too hard to change my habits.	
10	I haven’t changed because I’m afraid this wouldn’t work.	
Factor 3: Interpersonal relations		
11	Making this change would be criticized by those around me.	
12	I would be letting certain people down if I made this change.	
13	I’m worried that my friends would disapprove if I made this change.	
14	If I made the necessary change, I would probably be embarrassed when others noticed what I was doing.	
Factor 4: Lacking knowledge		
15	There’s so much information out there that I am confused about how to make this change.	
16	I don’t understand enough of the details about how to make this change.	
17	I’d like to change but I’m not sure where to begin.	
24	It’s the government’s responsibility to regulate this change.	
Factor 5: Tokenism		
18	The pro-environmental efforts that I currently engage in make further changes unnecessary.	
19	I’ve already made sacrifices to solve environmental problems, so there is no need for me to do more.	
20	I previously have made important effort in this, so there is no need for me to make further changes.	
21	My environmental actions already make enough of a difference.	
22	It’s not fair for me to change when really, it’s industry that’s causing the majority of environmental problems.	
23	The government should make it easier for me to change, if it really has the best interest of the environment in mind.	

Adapted—Psychological barrier factors and items		α
Factor 1: Conflicting goals and unnecessary changes		0.76
7	I believe that these changes would take too much of my time.	
10	I will not change because that would not work.	
2	I do not need to change because humans are not capable of saving planet Earth.	
8	I already invested too much on my way of living, thus I cannot change.	
1	It makes no sense in changing because technology is going to solve this problem in the future.	
Factor 2: Interpersonal relations		0.72
14	I would be embarrassed when others noticed that I have changed.	
11	If I change, I would be criticized by people around me.	
13	I am concerned that my friends would not approve if I changed.	
Factor 3: Conflicting goals and lacking knowledge		0.58
9	These issues are important for me, but it is too difficult to change my habits.	
17	I would like to change, but I really do not know where to start.	
15	I get confused about how to make this change because there is too much information around.	
Factor 4: Tokenism		0.73
20	I have already made many sacrifices for the environment, and now I do not need to do anything.	
21	I have already did too much in the past, not making it necessary to change even further.	
19	What I do for the environment is enough.	
Factor 5: Tokenism toward the government		0.66
23	It is not fair that I have to change, considering that major environmental problem causes are from industries and big companies.	
24	The government should facilitate this change if it really is concerned about the environment.	
18	The responsibility of commanding this change is from the government.	

### Study 2—Dengue’s questionnaire test results

**Figure 2:**
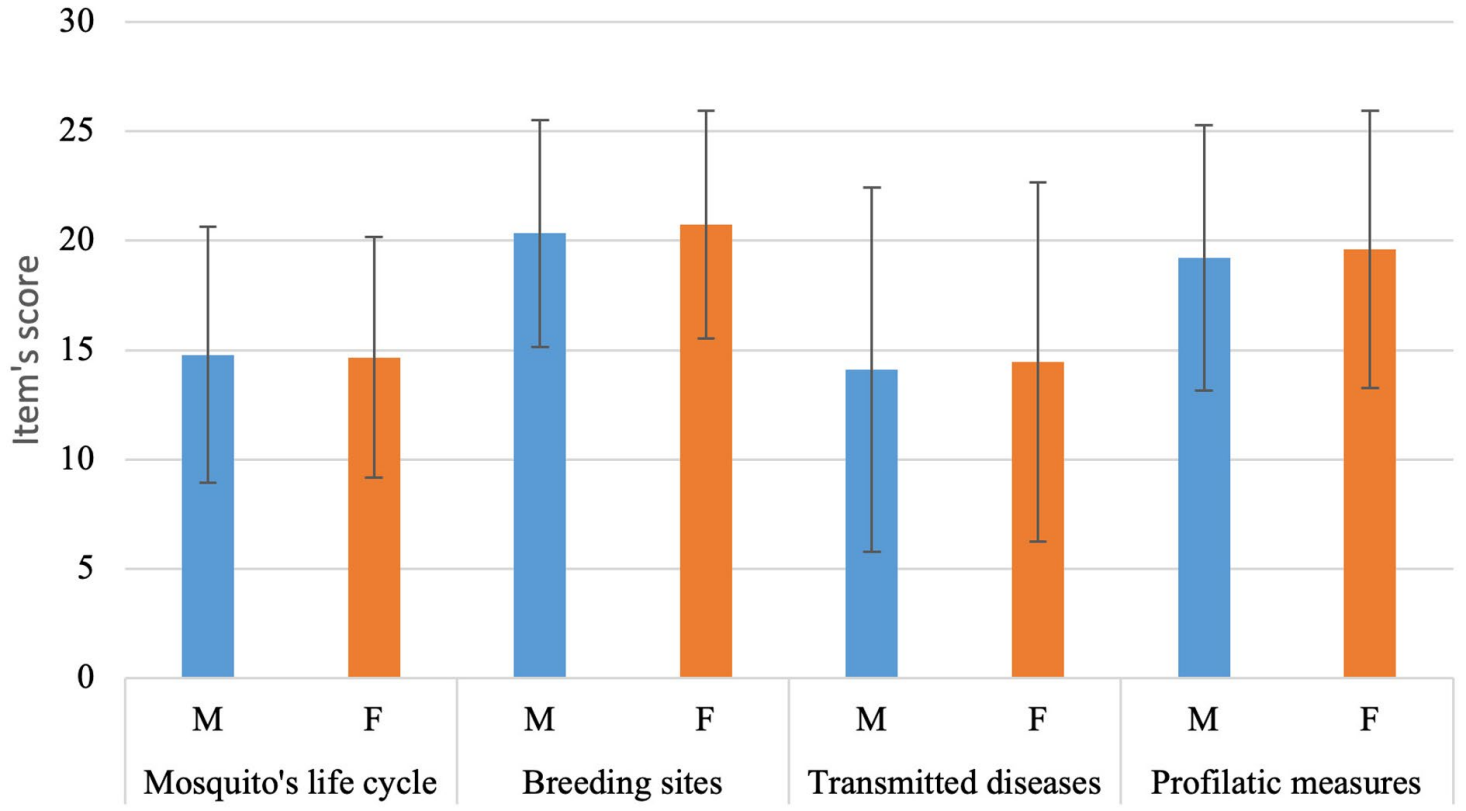
Average score for each of the four items in the Dengue fever’s test split into male (M) and female (F) (i.e., separated by the topics each referred to).

In total, the mean score was 68.95 and 70.8 median. Minimum score was 25 (*n* = 1) while maximum score was 100 (*n* = 10). Scores were grouped within quartiles, where first quartile was 58.3 (*n* = 109), second quartile was 70.8 (*n* = 121), third quartile was 79.10 (*n* = 128), and fourth quartile (*n* = 91) (Figure 2).

Item 01’s average score was 14.72 and 94 students achieved maximum score (20.93%). Although students were able to easily identify an adult mosquito from an egg, they struggled to differentiate larvae and pupae stages, thus reducing the item’s score (*n* = 337, 75.05%).

Item 02’s average score was 20.54 and 221 students achieved maximum score (49.22%). Items correctly related to mosquito’s reproduction that were most identified by students were leaving the water tank open (*n* = 435, 96.88%), plant vases (*n* = 388, 86.41%), disposed tires (*n* = 376, 83.74%), and rubbish incorrectly disposed (*n* = 337, 75.05%). At the same time, some actions that are not related to the mosquito were also mentioned as to be possibly related to it, like leaving the water tank closed (*n* = 32, 7.12%), rats and mice (*n* = 27, 6.01%), sneezing (*n* = 15, 3.34%), and holding hands (*n* = 6, 1.33%).

Item 03’s average score was 14.28 and 111 students achieved maximum score (24.72%). While most students were able to identify Zika (*n* = 352, 78.39%) and Chikungunya (*n* = 360, 80.17%) as possible diseases to be transmitted by *Aedes aegypti*, a smaller number was able to identify Yellow fever (*n* = 276, 61.46%). At the same time, other diseases, not related to *Aedes aegypti* were also mentioned as to be possibly transmitted by the mosquito, as in the flu (*n* = 149, 33.18%), AIDS (*n* = 60, 13.36%), COVID (*n* = 17, 3.78%), tetanus (*n* = 16, 3.56%), and diabetes (*n* = 10, 2.22%).

Item 04’s average score was 19.39 and 189 students achieved maximum score (42.09%). While most students were able to identify the importance of leaving the water tank closed to prevent mosquitos laying eggs (*n* = 432, 96.21%), other options were also mentioned, like avoiding water accumulation (*n* = 402, 89.53%), using sand on water vases (*n* = 327, 72.82%), and proper trash disposal (*n* = 298, 66.36%). At the same time, some behaviors that are not related to prevent mosquito’s reproduction were also selected by students, like getting in contact with rusted objects (*n* = 31, 6.9%), wearing a mask (*n* = 23, 5.12%), avoid hugging people (*n* = 15, 3.34%), sugar consumption (*n* = 11, 2.44%), and physical activities (*n* = 4, 0.8%).

There was no significant difference between schools on the total result (Kruskal-wallis, *p* = 0.9513). When evaluating items separately, no difference could be observed between different schools as well (item 01 *p* = 0.3266, item 02 *p* = 0.729, item 03 *p* = 0.4692, item 04 *p* = 0.8303). The total result also did not differ between genders (Kruskal-Wallis, *p* = 0.2614).

### Study 2—Confirmatory Factor Analysis (CFA)

**Figure 3:**
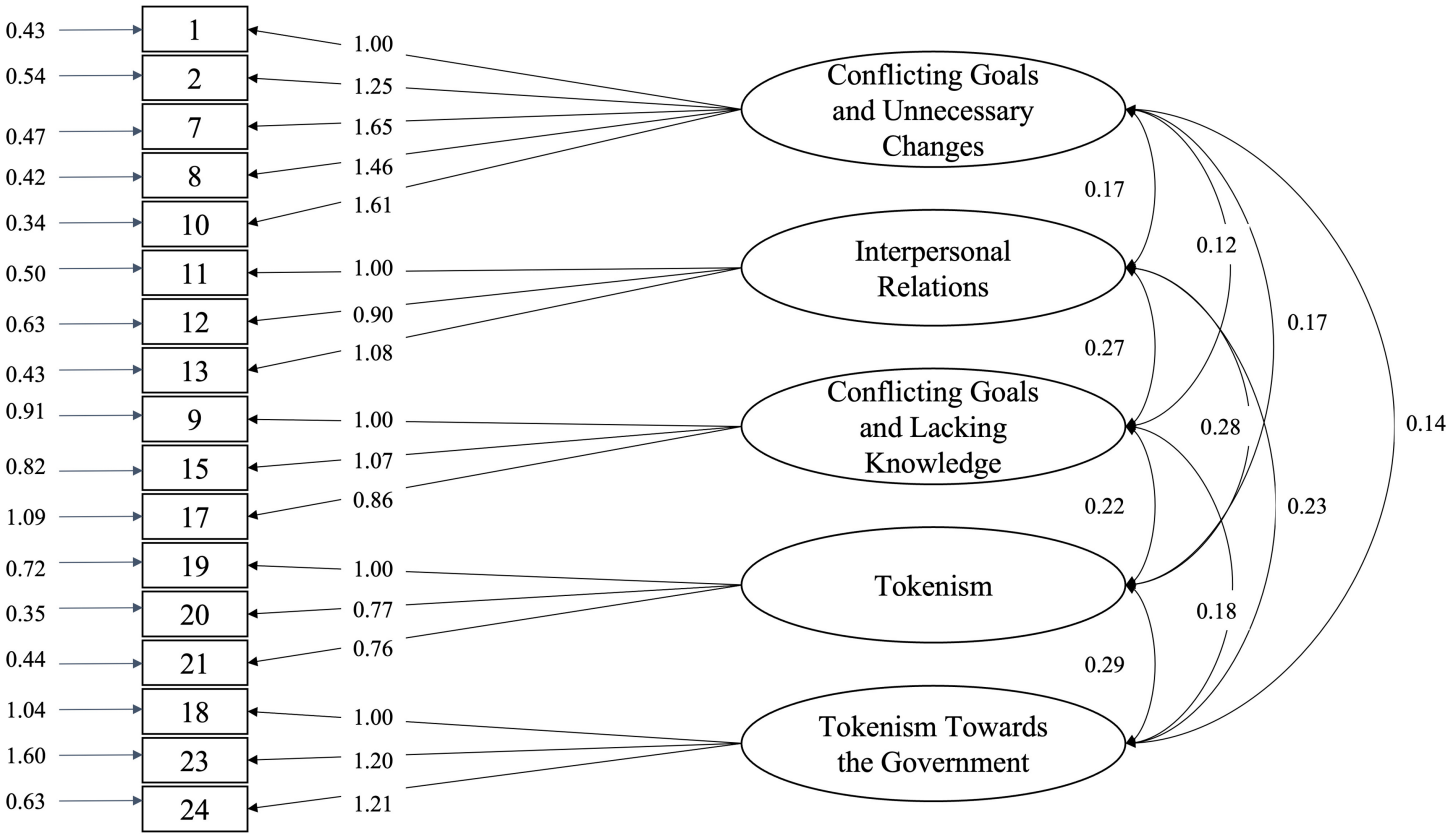
Five-factor model after item removal (*n* = 449). Arrows pointing from factors to items show factorial loadings, while arrows between factors show correlation, and arrows to the left, pointing to items show their error terms.

Confirmatory factor analysis was conducted to verify if items were consistent to its factors. Several indices were used to evaluate model fit (Table 3). Factorial loadings indicate that most variables have a strong association with its respective factors, while correlation between factors suggest a moderate relation and a good distinction between them (Figure 3).

**Table 3:** Model fit criteria for the five-item model after CFA.

Model	Model fit indices			
	*χ*^2^/df	RMSEA	CFI	GFI
Five-factors model	2.081	0.052	0.928	0.941

The table presents (1) chi-square to the degrees of freedom ratio (*χ*^2^/df, a ratio between 2 and 3 is considered is indicative of a good data-model fit), (2) Root Mean Square Error of Approximation (RMSEA, values close to 0.05 indicate a good fit), (3) Comparative Fit Index (CFI, values above 0.90 indicate a good fit), and (4) Goodness of Fit Index (GFI, values close to 0.95 are considered adequate).

**Figure 4:**
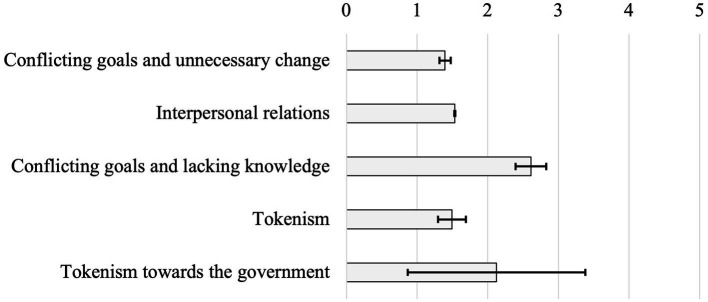
Mean barrier agreement’s scores for each of the analyzed factors (*n* = 449).

The total data collected by the DIPB scale initially pointed those items from factors 3 and 5 showed a higher level of agreement by students (Figure 4).

This pattern was maintained when analyzing the categories individually (i.e., schools, gender, and Dengue’s test result), with significant differences being presented below.

Data was initially split into four categories based on Dengue’s test results (i.e., low score, medium score, high score, and top-high score). Students with a low or medium score on the Dengue’s test presented a higher level of agreement for factors 1 and 2 (Table 4).

**Table 4:** Means and standard deviation (SD) of agreement for each factor based on Dengue’s test results.

		Factor 1	Factor 2	Factor 3	Factor 4	Factor 5
Total	Mean	1.3877	1.5208	2.6003	1.4823	2.1269
	SD	0.0184	0.0285	0.0346	0.0268	0.0381
Lower score	Mean	1.422	**1.7553**	2.6758	1.5259	2.1376
	SD	0.0374	0.0664	0.0681	0.0542	0.0727
Medium score	Mean	**1.4743**	**1.4352**	2.5785	1.5509	2.1487
	SD	0.0369	0.0467	0.0619	0.0524	0.0677
High score	Mean	**1.3421**	**1.513**	2.6.223	1.4.843	2.1901
	SD	0.0291	0.0491	0.0627	0.0466	0.0715
Top high score	Mean	**1.3296**	**1.4322**	2.5567	1.3919	1.9853
	SD	0.0369	0.0548	0.0746	0.0496	0.0757
Low vs. high		–	**	–	–	–
Medium vs. high		*	–	–	–	–
Top high vs. high		–	–	–	–	–
Medium vs. low		–	***	–	–	–
Top high vs. low		–	**	–	–	–
Top high vs. medium		*	–	–	–	–

Low score = <60.35; Medium score = between 60.35 and 70.8; High score = between 70.8 and 79.1; and Top-high score = above 79.1 and Tukey’s comparison between groups. Values that are in bold presented significant differences when compared to other groups (i.e., *p < 0.05; **p < 0.01; ***p < 0.001).

There was no significant difference between male and female answers, with a subtle tendency for factor 4 (*p* = 0.0664). Inside this factor, item 19 showed significant difference (*p*-value: 0.0464, male mean: 1.8984, male SD: 0.0829, female mean 1.6857, female SD: 0.0677), item 20 and 21 showed no significant difference.

Comparison between schools showed significant difference for factors 2 and 3 between school number two when compared to school number three (factor 02: **, factor 03), four (factor 02: **), five (factor 02: *), six (factor 02: **, factor 03: **), and seven (factor 02: *, factor 03: *) (Table 5).

**Table 5:** Means and standard deviation (SD) of the five factors for each of the 11 schools.

School		Factor 05	Factor 04	Factor 03	Factor 02	Factor 01
#1	Mean	2.192	1.494	2.750	1.667	1.438
	SD	0.103	0.066	0.098	0.092	0.048
#2	Mean	2.000	1.561	**2.982**	**1.860**	1.547
	SD	0.121	0.100	0.120	0.124	0.077
#3	Mean	2.271	1.479	2.500	**1.344**	1.313
	SD	0.150	0.102	0.130	0.071	0.056
#4	Mean	2.180	1.573	2.627	**1.373**	1.320
	SD	0.106	0.080	0.102	0.057	0.045
#5	Mean	1.951	1.486	**2.528**	**1.458**	1.388
	SD	0.106	0.079	0.100	0.086	0.060
#6	Mean	2.348	1.338	**2.453**	**1.433**	1.352
	SD	0.102	0.054	0.082	0.060	0.046
#7	Mean	2.000	1.362	**2.333**	**1.348**	1.330
	SD	0.149	0.090	0.114	0.085	0.061
#8	Mean	2.194	1.431	2.514	1.556	1.392
	SD	0.161	0.108	0.134	0.122	0.071
#9	Mean	1.935	1.529	2.710	1.623	1.457
	SD	0.110	0.088	0.109	0.097	0.062
#10	Mean	2.049	1.605	2.457	1.519	1.296
	SD	0.133	0.123	0.124	0.111	0.057
#11	Mean	2.103	1.603	2.706	1.667	1.462
	SD	0.110	0.083	0.113	0.094	0.056

Values that are in bold presented significant differences when compared to school #2 (i.e., *p < 0.05; **p < 0.01; ***p < 0.001).

For factors three, four and five, items 17, 19, and 24 were analyzed separately, due to a higher level of agreement when compared to other items within its own factor. Each of these items showed significant difference (*p* < 0.001) when compared to the other two.

## Discussion

### Study 1—Exploratory Factor Analysis

For the EFA, the present study identified a rearrange of the items and elimination of five of them. This variation may be explained by three different reasons. First, previous studies that validated the DIPB scale used a sample of undergraduate students, while here respondent’s age mean was 12 years old. This may affect how they interpret the items and connect to them (Boateng et al., 2018; Chwalow, 1995). Secondly, how people relate to the *Aedes aegypti* and Dengue fever situation is something quite specific for the countries that need to address this issue (Gubler and Clark, 1996). Third, different languages may not encapsulate the complete original meaning of the sentence, in which some subtle differences may affect the final interpretation of the item (Mager et al., 2018; Malá, 2013; Xian, 2008). Thus, although the DIPB scale was originally developed to be used for different ecological contexts, some adaptations may be needed depending on the target group and ecological context that is being analyzed.

### Study 2—Dengue fever test results

Many papers have been made trying to propose new approaches toward environmental education actions to fight the mosquito (Espinoza-Gómez et al., 2002; Fernandes et al., 2023; Lloyd et al., 1994).

By the answers given on the test, students showed that they are able to recognize situations and actions that are related to mosquito’s reproduction. The obtained data confirms what was expected by literature, where students understand what are the basic underlying mechanisms behind *Aedes aegypti*’s reproduction and how to stop it (Cruz and Santana, 2017; Silva et al., 2018). No significant difference was observed between schools, suggesting that the environmental actions that are being taken to educate the population are distributed homogenously.

Although these actions may present positive results, they are still not enough to control mosquito’s proliferation (de Souza et al., 2021). One key aspect that may justify this scenario and has been perceived in recent literature is the conflict between people’s and government’s responsibilities (lack of maintenance of public spaces by the government and lack of management on local dumpsters, for example) (Chiaravalloti et al., 2002; Donalisio et al., 2001; Heymann and Dar, 2014; de Oliveira and Lima, 2013; Rangel-S, 2008; Reis et al., 2013).

The two items that presented the lowest score were Items 01 and 03. The main reason for the first one was that students were not able to differentiate larval and pupal stages. Even though this reduced the item’s mean, it is hypothesized that it would not represent a meaningful difference on how people act to prevent mosquito’s reproduction. Mainly because both stages, larvae and pupae, share the same habitat (i.e., stagnant water).

Regarding Item 03, many students believe that the mosquito may transmit diseases like the flu and AIDS. This information may be relevant for designing future educational campaigns, both for preventing *Aedes aegypti*’s reproduction and Sexually Transmitted Infections (STI’s).

Items 02 and 04, related to the prophylactic measures necessary to reduce mosquito’s population and identifying areas where females lay their eggs presented the highest scores. This suggests that the educational actions taken by the government and schools are capable of addressing these topics to a younger audience.

### Study 2—DIPB’s results

Based on the DIPB’s scale, results suggest that current educational campaigns seem to be unable to address all analyzed barriers. Students that scored higher on Dengue’s test seemed to present less barriers toward adopting PEB for factors 01 and 02 (i.e., Conflicting goals and unnecessary changes and Interpersonal relations), but no significant difference could be observed for the other factors. Thus, teaching about the mosquito’s life cycle and what has to be done to prevent its proliferation (e.g., avoiding the accumulation of clean water in multiple spaces, like flower plates, vases, bottles, roof gutter, and garbage disposal) seems to not be enough to possibly change the behavior of some participants, considering that some psychological barriers are not being considered.

Overcoming these barriers seem to be the greatest challenge faced nowadays. The K&A model (Kollmuss and Agyeman, 2010) was created to identify internal (i.e., Personality traits and value system) and external factors (i.e., Infrastructure, Political, Social, and Cultural factors, and Economic situation) that may impose barriers to PEB. This model is being tested in recent years (Graves and Roelich, 2021) and some of these factors could justify the barriers identified in this study.

Factors 03 and 05 (i.e., Conflicting goals and lacking knowledge and Tokenism toward the government) presented the highest level of agreement by students overall. The items that presented the higher level of agreement were 17 (factor 3, mean: 3.0098, *I would like to change, but I really do not know where to start*) and 24 (factor 5, mean: 2.6683, *the government should facilitate this change if it really is concerned about the environment*). Considering the presented results, and what has been described in previous papers, it seems to be imperative that educational programs continue to address and reinforce PEB by the population, but also facilitates the communication to the local government, making it clear the responsibilities between internal and external factors. It is proposed that this communication needs to address public spaces management and general complaints.

Wu et al. (2023), states the relevance of local governments in regulating and empowering the population, mainly by resource mobilization and political processes for residents to manifest desired PEB. Other studies have also identified how the role of perceived government is able to influence PEB (Berglund and Matti, 2006; Lavergne et al., 2009).

School # 2 presented higher values for factor 2 and 3, presenting a significant difference when compared to other schools, even though there was no significant difference this particular institution and other schools for the Dengue’s questionnaire. The school is located in the biggest city’s neighborhood, attending students from a variety of regions. This result may be explained by the access those students have to educational campaigns from the government and media, but more studies are necessary to understand this variation. To better understand it, a multi-variable analysis is suggested, where aspects like (1) government’s presence in the region (i.e., parks maintenance and waste disposal), (2) frequency of sanitary agents visits, and (3) socioeconomically aspects (Corral-Verdugo and Pinheiro, 1999; de Souza et al., 2021) are suggested and may be responsible for eliciting these psychological barriers.

This pattern repeated itself even with students that scored high on Dengue’s test, showing that they understand what is necessary to inhibit mosquito’s reproduction, but maybe do not feel in the position to take action. Therefore, environmental education programs and actions need to address these barriers to encourage students to take action to protect the environment and prevent the proliferation of Dengue mosquitoes.

### Suggestions for future environmental education projects

Environmental education projects and actions developed in the last decades have distanced themselves from a conservationist point of view to a more critical perspective. In a research on developed papers from between 2003 and 2007, (Pato et al., 2009), identified that a socioenvironmental approach, that recognizes political and cultural aspects as part of a new epistemological perspective is notable. Thus, creating new to ways to build positive connections between the population and natural environmental seems to be the current tendency (Ardoin et al., 2020; Li et al., 2024; San Jose and Nelson, 2017).

When considered the actions toward inhibiting Dengue mosquitoes’ reproduction, a practical approach is, in many cases, adopted (Luz et al., 2024; Rosa et al., 2020). One of the reasons for that is the large scale in which this subject is engaged, through the television, radio, educational campaigns, and others. These procedures repeat themselves in different places, with almost no change on how they occur.

In these strategies, the participant, a mere receptor of information, must replicate the instructions in their houses or region where they live. The change on how they relate to the environment, government, and how information and experiences are exchanged is mostly disconsidered. It is believed that one of the main reasons for that is the use of indicators to measure the efficiency of these campaigns. The term efficiency is used on this study because of the relation between educational campaigns and reduction of cases reported or breeding sites identified by public agents (Arantes et al., 2023; Caregnato et al., 2008; Gonçalves et al., 2023; Luz et al., 2024; Sulistyawati, 2024).

Thus, it is initially suggested a segmentation between the different goals an environmental education project or action on that matter might have. While some actions may focus in informing about the mosquito, symptoms and how to prevent its proliferation (dos Santos et al., 2023), others must focus on creating more powerful connections between the population, environment and government (Busato et al., 2024; Martín et al., 2024; de Oliveira and Lima, 2013; de Souza Pinto et al., 2013). By doing that, and clearly establishing the responsibilities between the many responsible agents of that phenomenon, it is expected to diminish the barriers identified in the studies here presented.

This seems to be general consensus of what has already been described in recent literature (Costa and dos Anjos Carneiro-Leão, 2021; dos Santos and Vieira, 2023) A recently published review on five decades of Dengue’s prevention and control in Singapore showed four key features that were responsible for significantly reducing the mosquito index, where coordinated inter-sectoral cooperation is one of them (Ho et al., 2023).

To inform the population about individual, collective, and governmental responsibilities, creating ways to connect those points may be an important step in reducing the barriers here presented. However, not only that, but also to find ways to create positive connections between different segments of society and nature, strengthening values and beliefs that are closely related to PEB’s is essential to create long lasting changes (de Lima and Pato, 2021; Portus et al., 2024; Tucholska et al., 2024; Goulart et al., 2016), points out that the lack of information, absence of environmental education campaigns, rapid proliferation of the mosquito, and lack of governmental preventive actions are among the main factors that weakens public policy, even in regions with adequate budgets to fight the disease. It is then perceived the environmental education programs cannot be reduced to isolated actions of informing the population, but something that creates a mosaic of actions and projects, with different goals, capable of aiming internal and external factors for various environmental challenges.

## Conclusion

This work aimed to identify possible psychological barriers behind the adoption of pro-environmental behaviors expected to reduce *Aedes aegypti*’s population. For that, a scale for psychological barriers was initially validated for Brazilian children, and then tested across multiple variables. This is the first study to assess psychological barriers toward *A. aegypti* using a quantitative methodology. Thus, considering the nature of an exploratory study and subject’s novelty, some considerations and propositions are made for future investigations. For future research, understanding if different target groups (i.e., age group, geographical location, education levels and others) present similar barriers, and comparing different strategies of environmental education projects and actions seems to be an important step to validate possible trends in psychological barriers toward PEB’s on that matter.

The DIPB scale had to be adjusted after EFA and some factors were rearranged. It is suggested that new adaptations may be needed to accommodate the subtle variables that are present in different geographical regions, linguistic aspects, and target group (i.e., age, education level, and economical background). Also, regional differences and the impact caused by *A. aegypti* infestation and its correlated diseases may affect the barriers observed in different regions.

Dengue’s questionnaire showed that students know what has to be done to prevent mosquito’s proliferation, and how water is connected to its life cycle. However, the diseases that may be transmitted by it, besides Dengue fever, still seem to be confusing.

Students that scored lower in Dengue’s questionnaire presented more barriers when compared to those who scored higher for factors 01 (Conflicting goals and unnecessary changes) and 02 (Interpersonal relations). Educational programs seem to be effective to reduce these barriers, even though some are still present.

On the adapted DIPB scale, students presented a higher agreement on items 17 (*I would like to change, but really do not know where to start*), 19 (*What I do for the environment is enough*), and 24 (*The government should facilitate this change if it really is concerned about the environment*). These results suggests that future educational campaigns should build different actions that focuses on addressing both internal and external factors, creating a mosaic of projects, with different goals, each aiming different environmental challenges.

## Data Availability

The raw data supporting the conclusions of this article will be made available by the authors, without undue reservation.
